# Chinese herbal medicine *bi min fang* for allergic rhinitis: protocol for a double-blind, double-dummy, randomized controlled trial

**DOI:** 10.1186/s13063-018-3151-0

**Published:** 2019-01-18

**Authors:** Qiulan Luo, Shiqing Zhou, Xiaoshan Li, Qubo Chen, Wenmin Lin, Liming Lu, Hua Li, Caifeng Chen, Wenyong Chen, Yunying Li

**Affiliations:** 1 Otorhinolaryngology Department, Second Affiliated Hospital of Guangzhou University of Chinese Medicine, 111 Dade Road, Yuexiu District, Guangzhou, 510120 Guangdong Province China; 2 Otorhinolaryngology Department, Guangdong Provincial Hospital of Chinese Medicine, 111 Dade Road, Yuexiu District, Guangzhou, 510120 Guangdong Province China; 3 The Second Clinical College of Guangzhou University of Chinese Medicine, Guangzhou, 510120 China; 4 Otorhinolaryngology Department, Guangdong Provincial Hospital of Integrated Traditional Chinese and Western Medicine (Nanhai District Hospital of Traditional Chinese Medicine of Foshan City), 16 Nanwu Road, Nanhai District, Foshan, 528200 China; 5 Affiliated Guangdong Hospital of Integrated Traditional Chinese and Western Medicine of Guangzhou University of Chinese Medicine, 16 Nanwu Road, Nanhai District, Foshan, 528200 China; 6 Biological Resource Center, Guangdong Provincial Hospital of Chinese Medicine, Guangzhou, 510120 China; 7 Clinical Research Center, South China Research Center for Acupuncture and Moxibustion, Medical College of Acu-Moxi and Rehabilitation, Guangzhou University of Chinese Medicine, Guangzhou, 510006 China

**Keywords:** Allergic rhinitis, Chinese herbal medicine, Randomized controlled trial, Cytokines, Gut microbiota

## Abstract

**Background:**

People with allergic rhinitis (AR) often seek help from Chinese medicine due to dissatisfaction with conventional treatments. Lung-spleen *qi* deficiency syndrome (LSQDS) is the most common type of AR, and the Chinese herbal medicine formula *bi min fang* (BMF) is commonly prescribed for AR patients with LSQDS. However, direct evidence supporting its efficacy and safety is not available, and its potential mechanism of action remains unclear.

**Methods/design:**

This paper presents a double-blind, double-dummy, randomized controlled trial. After a 2-week run-in period, 80 AR patients with LSQDS will be recruited and randomly allocated to the BMF group or the control group in a 1:1 ratio. The patients in the BMF group will receive BMF and the placebo for levocetirizine hydrochloride orally, while the control group participants will receive levocetirizine hydrochloride and the placebo for BMF orally. All participants will receive 4 weeks of treatment and 12 weeks of follow-up.

The primary outcome is a change in the Total Nasal Symptom Score (TNSS). Secondary outcomes include changes in scores for the standard version of the Rhinoconjunctivitis Quality of Life Questionnaire (RQLQ(S)), and visual analog scale (VAS); changes in serum levels of the cytokines interleukin-4, interferon-γ, transforming growth factor β-1, and interleukin-17; and changes in the gut microbiota composition in the stool. The TNSS, RQLQ(S), and VAS will be recorded at the beginning of, middle of and after the treatment period and at the end of each month in the 3-month follow-up period. Blood and stool samples will be collected at baseline and the end of the treatment. The aforementioned four cytokines will be detected in the serum using enzyme-linked immunosorbent assays, and the stool gut microbiota will be detected using 16S ribosomal ribonucleic acid sequencing. Any side effects of the treatment will be recorded.

**Discussion:**

The results of this trial will provide consolidated evidence of the effect of BMF on AR and the potential mechanism by which BMF acts. This study will be the first to explore the mechanism of action of Chinese herbal medicine on the gut microbiota in AR.

**Trial registration:**

Chinese Clinical Trial Registry, ChiCTR-IPR-17010970. Registered on 23 March 2017.

**Electronic supplementary material:**

The online version of this article (10.1186/s13063-018-3151-0) contains supplementary material, which is available to authorized users.

## Background

Allergic rhinitis (AR) is a symptomatic nasal disorder caused by an immunoglobulin E (IgE)-mediated immunological reaction to allergen exposure [1] and is a global health problem that affects people of all ages. The worldwide incidence of AR is 10–20% [2], and the prevalence of self-reported AR in China is 11.1–19.1% [3, 4]. Classic symptoms of AR include rhinorrhea, nasal obstruction, nasal itching, and sneezing [1], and the disorder is classified as intermittent AR or persistent AR based on the duration of symptoms [1]. AR can lead to sleep cycle disorders, emotional imbalance, impaired ability to perform normal daily activities, severely decreased quality of life, and significant economic burden [1, 5]. Moreover, AR also serves as a trigger for other diseases, such as bronchial asthma [1]. Current conventional management of AR primarily includes allergen avoidance, pharmacotherapy, immunotherapy, and patient education [1, 6]. Second-generation H1 antihistamines, nasal glucocorticosteroids and leukotriene antagonists are recommended as the first-line therapy [1, 2, 6]. However, due to unsatisfactory results with conventional treatment, AR patients are increasingly seeking complementary and alternative therapies [7].

Chinese herbal medicine (CHM) is a well-tolerated choice for AR patients seeking complementary and alternative therapies to reduce AR symptoms [7]. The latest Chinese guidelines for the diagnosis and treatment of allergic rhinitis (2015, Tianjin) suggest that CHM can be applied as an auxiliary method of treating AR [4]. CHM is an important type of complementary alternative medicine, and several studies have focused on the use of CHM to treat AR. A double-blind randomized controlled trial (RCT) with a design incorporating repeated measures and three parallel groups showed that CHM is useful for ameliorating symptoms, enhancing quality of life, and strengthening body constitution in patients with AR, and all participants in this study were subjected to syndrome differentiation [8]. Another two RCTs showed that CHM safely reduces the nasal symptoms of AR, although the participants in those studies were included without syndrome differentiation [9, 10]. One systematic review and meta-analysis of seven RCTs comparing oral CHM to a placebo showed that CHM was able to reduce total nasal symptom scores. However, details of the CHM formulas used in the studies included were not investigated, and the syndromes of the participants were not described. Furthermore, the sample sizes of the RCTs were small, and all the trials had certain methodological limitations. Thus, the authors could not draw a firm conclusion regarding the effects of CHM on AR [11]. Overall, CHM appears to be a promising intervention for patients with AR, and more rigorous RCTs with large sample sizes are needed to further define its effectiveness.

Syndrome differentiation or pattern identification is the core treatment principle of traditional Chinese medicine (TCM), and an accurate treatment for an AR patient must be prescribed according to their body constitution. Based on our clinical observations, a considerable number of AR sufferers are diagnosed with lung and spleen *qi* deficiency syndrome (LSQDS), and this observation is supported by the results of published studies [12–16]. Therefore, according to TCM theory, tonifying lung and spleen *qi* is the treatment principle for AR patients with LSQDS.

Two classic CHM formulas, *bu zhong yi qi tang* and *yu ping feng san*, are commonly used for allergic diseases [17]. In our clinical practice, *bi min fang* (BMF), which is composed of modified *bu zhong yi qi tang* and *yu ping feng san*, is the most commonly used CHM formula for AR patients with LSQDS. The composition of BMF is based on a systematic review of classic reference books, articles obtained from the PubMed database [8, 17], the Chinese scientific literature, clinical guidelines [18], and our clinical experience [19].

Existing evidence supports the beneficial effects of two original TCM formulas on AR [8, 20–22]. Clinical studies have shown that *yu ping feng san* can improve symptoms [8, 21] and quality of life [8] and decrease the levels of interleukin-4 (IL-4) and IgE in AR patients [21]. Indeed, a glucosidic extract from *yu ping feng san* reportedly exerts anti-inflammatory and immunoregulatory effects by inducing activation of T helper cells and regulating other subsets of T lymphocytes [22]. *Bu zhong yi qi tang* was also found to reduce AR symptoms, with suppressive effects on the total serum level of IgE and IL-4-stimulated production of prostaglandin E2, leukotriene C4, and COX-2 mRNA expression in IL-4-stimulated polymorphonuclear neutrophils [20]. Thus, it is reasonable to hypothesize that BMF is effective for the treatment of AR.

Most CHM is administered orally, whereby formulations are ingested, transferred, digested, and absorbed through the gastrointestinal system. Accordingly, CHM may influence the gastrointestinal system, including the intestinal mucosa and gut microbiota, the latter of which is essential for health and closely linked to diseases [23]. For instance, several studies have demonstrated that low gut microbiota diversity is associated with a high risk of allergic diseases [24–28]. Other studies have reported that the characteristics of seasonal AR include a lower diversity of intestinal *Bifidobacterium* [29], with a reduction of *Bifidobacterium*, *Clostridium*, and *Bacteroides* [30] and increased populations of *Bacteroides fragilis* [31]. A few systematic reviews and studies indicate that certain probiotics are beneficial for patients with AR [32–35]. We hypothesize that components of CHM, such as glycosides and oligosaccharides [36], may influence the gut microbiota in the same manner as an oral probiotic by regulating local intestinal immunological conditions and thereby, achieving systematic immunomodulation [37, 38].

Placeboes have been widely used in the control groups of previous clinical studies of AR treated with pharmacotherapy and immunotherapy, with the placebo control treatment resulting in over 50% symptom relief [6]. Therefore, in this study, we selected Western medicine as the positive control instead of a placebo. Levocetirizine, a second-generation antihistamine that acts on peripheral histamine H1 receptors, has a strong anti-allergic effect without a sedative effect, and it is used for relieving and preventing not only AR symptoms but also those of other allergic diseases. After careful consideration of the adverse effects and the desired effect, we chose levocetirizine as the intervention for the control group.

To blind the participants and the researchers adequately, a placebo for the intervention given to the other group will be administered to both groups. We designed a double-blind, double-dummy RCT for AR patients with LSQDS. The objectives are to assess the efficacy of BMF for AR patients with LSQDS and to explore the mechanism of BMF in treating AR with respect to the immune response and gut microbiota.

## Methods/design

### Study design and setting

**Fig. 1:**
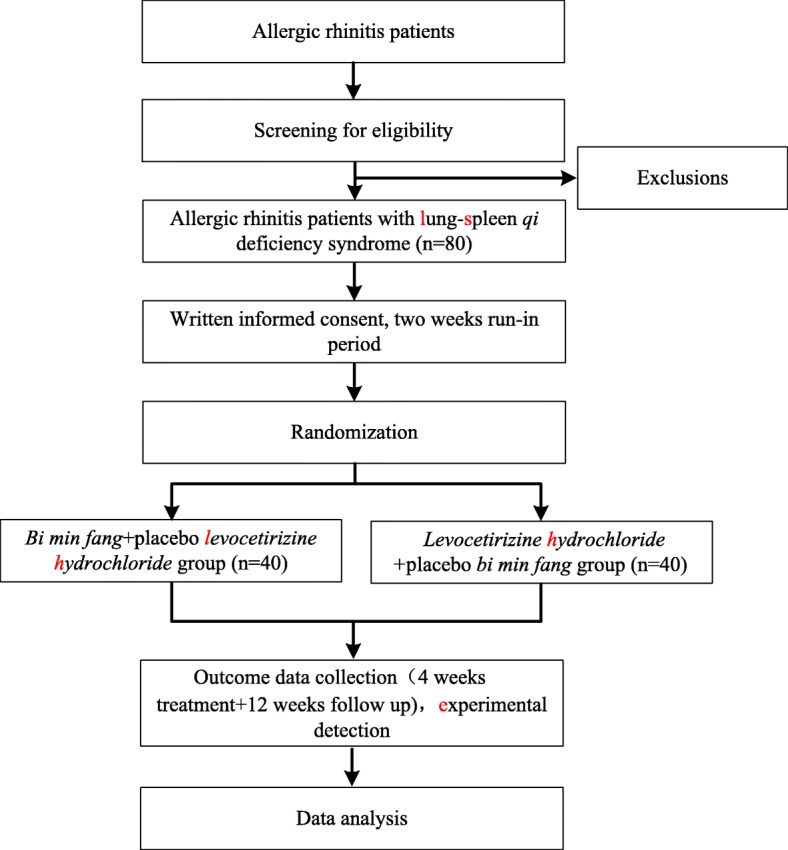
Trial flow chart

**Fig. 2:**
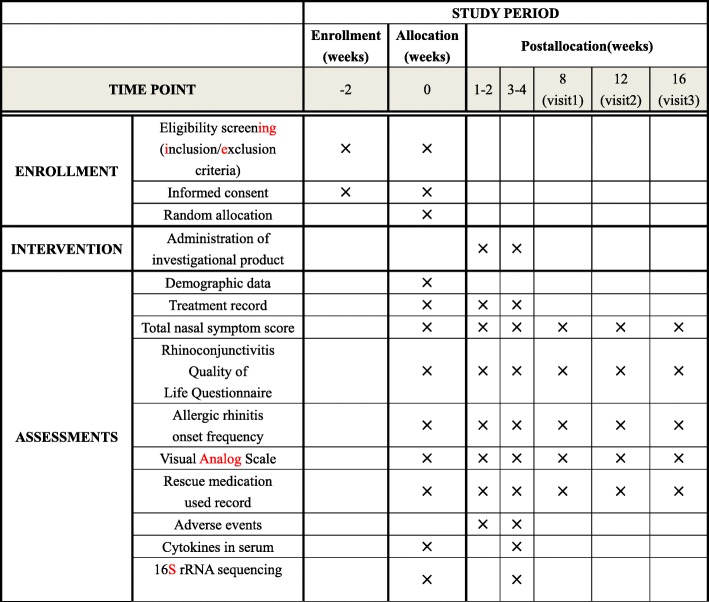
Study period

This is a double-blind, double-dummy RCT. In total, 80 participants will be recruited from the Guangdong Provincial Hospital of Chinese Medicine (GDPHCM), Guangzhou, China. The flow chart and study period are shown in Figs. 1 and 2, respectively. After obtaining written informed consent, a 2-week run-in period will be implemented, and then eligible participants will be randomly assigned to a CHM group or a control group in a 1:1 ratio. Because anther study designed to identify differences in the gut microbiota between AR patients and healthy controls will utilize stool samples from the same group of AR participants, additional consent provisions for collection and use of these participants’ data and stool samples will be explained to the participants.

### Participants

Participants will be recruited via a local advertisement and doctor referrals from otorhinolaryngology clinics at GDPHCM. Information about the study, processing, and scheduling will be carefully explained before enrollment. Participants must meet the Western medicine diagnostic criteria for AR (Table 1) [4] and the TCM syndrome diagnostic criteria for LSQDS (Table 2). Syndrome differentiation will be determined by two independent qualified TCM otolaryngologists. Participants can complete their case report forms (CRFs) independently, and blood and stool samples can be collected according to the instructions.

**Table 1:** Western medicine diagnostic criteria for allergic rhinitis

Symptoms	Two or more symptoms such as rhinorrhea, nasal obstruction, nasal itching, and sneezing, persisting for a cumulative period greater than 1 h per day. These symptoms may be accompanied by itchy and red eyes and tears.
Signs	The nasal mucosa is pale and edematous. Nasal secretions are watery.
Allergen test	At least one type of skin prick test or serum-specific IgE test is positive.

**Table 2:** Diagnostic criteria for TCM differentiation of lung and spleen *qi* deficiency syndrome

History	Participants may have a personal or family history of allergies.
Clinical symptoms	The clinical symptoms are characteristically sudden and repeated. The main symptoms of AR are nasal itching, paroxysmal sneezing, watery rhinorrhea, and nasal congestion. These symptoms may be accompanied by itchiness of the eyes, pharynx, or soft palate.
Signs	The nasal mucosa is pale gray, pale blue, or red. The nasal turbinate is congested, and the nasal cavity is filled with a watery discharge. All these signs are unapparent when in remission. A light pink tongue body with a thin white coating or teeth marks on the sides and a thin and weak pulse are also characteristic.
Other laboratory tests	Immunological tests such as a skin prick test and serum IgE test are helpful in making a diagnosis.

*AR* allergic rhinitis, *TCM* traditional Chinese medicine

### Eligibility criteria

Inclusion criteria:

- In accordance with the Western medicine diagnostic criteria for AR. Presence of persistent AR and meeting the criteria for lung and spleen *qi* deficiency in TCM
- Age 18 to 65 years old, with no sex limitation
- Has not taken hormones or antibiotics in the preceding 3 months and has not consumed yogurt or prebiotics for 1 month preceding the trial
- Participants provided informed written consent and volunteered to participate in the study.
- Participants can complete the case report forms (CRFs) independently, and blood and stool samples can be collected according to the instructions.

Exclusion criteria:

- Patients with similar nasal diseases such as acute rhinitis, vasomotor rhinitis, autonomic nervous rhinitis, eosinophilia nonallergic rhinitis, and allergic sinusitis
- Pregnant women. The trial will be suspended if a participant becomes pregnant
- Participants who cannot fully cooperate with the trial due to mental or behavioral disorders
- Participants who frequently change their work place, resulting in difficulty with follow-up
- Participants who drive or work high above the ground

### Randomization and allocation concealment

Randomization will be performed by the Key Unit of Methodology in Clinical Research of GDPHCM. The PROCPLAN process statements of the SAS statistics and analysis system (SAS Institute Inc., Cary, NC, USA) will be used to generate random numbers, which will then be enclosed in opaque envelopes. Attention will be paid to the concealment of the randomization. Eligible participants will be randomly assigned to either the BMF group or the control group.

Treatment allocation will be concealed and held by one member of the research team. To ensure adequate concealment, the participants will be given sequential treatment cards by independent researchers. The participants will receive an opaque envelope and allocated to one of the two groups according to the serial number and group name printed on their treatment card.

### Blinding

The research team members, with the exception of the clinical research methodology personnel and eligible participants, will be blinded to the treatment allocation. The study code will not be revealed until the end of the study, unless there is a serious adverse event (AE).

This study is a double-blind, double-dummy RCT, and the medicine used for treatment and the placebo will be identical in appearance. In addition, the research team will be instructed not to communicate with the participants regarding their possible treatment group allocation.

### Interventions

The participants in the CHM group will be orally given BMF compound granules (30 mg twice daily) and a placebo for levocetirizine (10 ml/5 mg every night) for 4 weeks. The participants in the control group will be orally given levocetirizine (10 ml/5 mg every night) and a placebo for BMF compound granules (30 mg twice daily) for 4 weeks.

The ingredients, dosage, and pharmacological action of BMF are presented in Table 3 [39]. The placebo for BMF compound granules will be composed of starch and an edible pigment and will be matched as closely as possible to the appearance and taste of BMF. The placebo for the levocetirizine oral solution has the same appearance and dosage as the levocetirizine oral solution but lacks the active ingredient. All interventions will be produced by the manufacturers following good manufacturing practices. The CHM compound granules and CHM placebo will be produced by Jiangyin Tianjiang Pharmaceutical Co., Ltd. (Jiangyin City, Jiangsu Province, China, Good Manufacturing Practice certificate number: JS20140328, batch number: 1608359). Levocetirizine oral solution and its placebo will be produced by Chongqing Huapont Pharmaceutical Co., Ltd. (Chongqing City, China, production license: CQ20130006, batch number: 20160801). All interventions meet the requirements of the regulatory guidelines issued by the China Food and Drug Administration.

**Table 3:** Composition and action of *bi min fang*

Ingredients	Percentage (%)	g/sachet	g/day	Action
Radix astragali (*huang qi*)	17.6	7.5	15	TCM: 1 Tonifying the spleen and lung *qi* and increasing *yang*. 2. Strengthening the defense *qi* and securing the exterior. 3. Inducing diuresis and removing edema Pharmaceutical study: 1. Enhancing the immune effect. 2. Inducing diuresis
Rhizoma atractylodis macrocephalae (*bai zhu*)	17.6	7.5	15	TCM: 1 Tonifying the spleen and replenishing *qi*. 2. Eliminating dampness and inducing diuresis. 3. Hidroschesis Pharmaceutical study: 1. Maintaining a strong constitution. 2. Enhancing immunologic function. 3. Inducing diuresis
Radix saposhnikoviae (*fang feng*)	11.8	5	10	TCM: 1. Dispelling wind and releasing the exterior. 2. Dispelling dampness and relieving pain. 3. Stopping convulsions Pharmaceutical study: 1. Antimicrobial and anti-inflammatory effects. 2. Analgesic and anticonvulsive effects
Radix paeoniae alba (*bai shao*)	11.8	5	10	TCM: 1. Nourishing blood and constraining *yin*. 2. Emolliating the liver and relieving pain Pharmaceutical study: 1. Analgesic effect. 2. Antimicrobial and anti-inflammatory effect. 3. Immunologic effect
Herba ecliptae (*mo han lian*)	11.8	5	10	TCM: 1. Enriching the *yin* of the kidney and the liver. 2. Cooling the blood and stopping bleeding Pharmaceutical study: 1. Enhancing immunity. 2. Protecting the liver. 3. Antimutagenic activity
Radix bupleuri (*chai hu*)	11.8	5	10	TCM: 1. Releasing the exterior and abating fever. 2. Soothing liver-*qi* stagnation. 3. Increasing *yang qi* Pharmaceutical study: 1. Inhibiting the central nervous system. 2. Anti-inflammatory and antimicrobial effects. 3. Enhancing immunologic function. 4. Protecting the liver
Xanthii fructus (*cang er zi)*	11.8	5	10	TCM: 1. Dispersing wind and cold. 2. Dispelling dampness. 3. Relieving nasal congestion. 4. Relieving pain Pharmaceutical study: 1. Relieving nasal symptoms. 2. Antimicrobial activity. 3. Preventing coughing
Radix et rhizoma glycyrrhizea (*gan cao*)	5.9	2.5	5	TCM: 1. Tonifying the spleen and replenishing *qi*. 2. Dispelling phlegm and suppressing coughing. 3. Relaxing tension and relieving pain. 4. Clearing heat and detoxifying. 5. Harmonizing medications Pharmaceutical study: 1. Protecting the digestive system. 2. Antiallergic and anti-inflammatory effects

If a participant cannot take the medication for any particular reason, a record will be made, and the medicine will be conserved and returned to the researchers at the end of the study. Two follow-up telephone visits will be performed during the treatment period. Participants who complete treatment will be followed up three times over the course of 3 months (Fig. 2). No other treatment for AR will be allowed for any participant in either group during the study period. The rescue medication, desloratadine tablets (5 mg orally every night, Hainan Poly Pharmaceutical Co., Ltd., Chinese State Food and Drug Administration approval number: H20040972), will be provided for participants who may experience AR episodes. Administration of the rescue medication will be recorded and statistically analyzed as an indicator of a curative effect.

### Outcome measurement

The primary outcome is a change in the Total Nasal Symptom Score (TNSS) [40]. Secondary outcome measures are as follows: (1) changes in the standard version of the Rhinoconjunctivitis Quality of Life Questionnaire (RQLQ(S)) score; (2) the frequency of AR episodes and their severity (visual analog scale, VAS); (3) changes in the serum levels of cytokines IL-4, interferon-γ (IFN-γ), transforming growth factor β-1 (TGF-β1), and interleukin-17 (IL-17), and changes in the gut microbiota composition in stools before and after treatment. In addition, participants will complete a medicine diary and report any AEs related to the intervention drugs throughout the trial.

The TNSS evaluates the following four nasal symptoms: rhinorrhea, nasal obstruction, nasal itching, and sneezing. The symptoms are self-assessed and recorded by subjects using a four-point scale (0 = no symptoms, 1 = mild symptoms, 2 = moderate symptoms, and 3 = severe symptoms), with low scores indicating less severe nasal symptoms [40]. The RQLQ(S) is a disease-specific questionnaire that can assess quality of life impairment in AR patients. This questionnaire consists of 28 questions covering the following seven domains: activities, sleep, non-nose/eye symptoms, practical problems, nasal symptoms, eye symptoms and emotional problems [41]. The RQLQ(S) with standardized activities is an updated version of the original RQLQ [42]. It has been translated into different languages and has frequently been used in different countries. The VAS, which ranges from 0 (nasal symptoms not at all bothersome) to 10 (nasal symptoms extremely bothersome) was designed to assess the severity of nasal symptom disturbance and has been validated for use in the quantitative evaluation of AR severity [43]. The TNSS, RQLQ(S), and frequency and severity of AR episodes will be evaluated at the beginning of, middle of and after the treatment period and at the end of each month during the 3-month follow-up period (Fig. 2).

Blood and stool samples will be collected before and after the treatment period (Fig. 2). The blood samples will be used to detect the serum levels of the cytokines IFN-γ, IL-4, TGF-β1, and IL-17 using enzyme-linked immunosorbent assays (ELISAs). These cytokines are typical inflammatory cytokines involved in immune disorders and are released by Th1, Th2, T-regulatory, and Th17 cells [44, 45]. To observe the influence of BMF on the composition of the gut microbiota, 16S ribosomal ribonucleic acid (16S rRNA) sequencing will be used to detect bacterial taxa present in stool samples.

A Health and Diet Habits Questionnaire, sampling instructions, a cardboard box, a sampling spoonand a fecal collection tube with cached liquid will be distributed to each participant before the stool samples need to be collected. All participants will be requested to complete the Health and Diet Habits Questionnaire, after which they will be asked to defecate after urinating. The stool will be collected in the cardboard box. The participants will use the sampling spoon to collect two spoonfuls of stool (approximately two grams) without having the sample contact the urine, sewage and fecal pool, and they will deposit the sample in the fecal collection tube. They will then tightly cover the tube and shake it until the sample has uniformly mixed with the cached liquid. The fecal collection tube will keep the stool fresh for 48 h at room temperature. As some participants cannot defecate in a hospital, they will take the above materials home and send their stool samples to the researchers by S.F. Express to arrive within 1 day. All blood samples will be sent to the Biological Resource Center of the GDPHCM within 2 h, and the stool samples will be sent within 24 h. All blood and stool samples will be stored at −80 °C. The 16S rRNA sequencing of the gut microbiota will be performed by BGI Shenzhen Co., Ltd.

Personal information will be collected from potential and enrolled participants by authorized researchers who will be trained at the beginning of the study. Personal information will not be shared without the participants’ agreement.

### Quality assurance and data management

To ensure strict adherence to the study protocol and familiarity with the trial administration process, an independent steering committee will be formed by the principal investigator prior to the beginning of the study. This committee will be composed of one independent chairman (Dr. Wenyong Chen), Dr. Hua Li, Dr. Caifeng Chen, and two other independent members, including at least one patient and a public involvement representative. The responsibilities of the steering committee will include approving any amendments to the main study protocol, monitoring the trial, and approving and commenting on project deliverables. The entire research team will then be provided with a standard operating manual detailing the procedures and will be required to undergo training.

All information from CRFs will be carefully recorded. All errors will need to be crossed out and corrected, after which the correction must be signed and dated by the participant or researcher. Patient withdrawals or missed follow-up visits will be recorded in the CRFs. An independent Data Monitoring Board will be established at the beginning of the study and will be composed of four fully independent members: a chairman, an otolaryngologist, a biostatistician and a clinical pharmacology expert from the GDPHCM. The Data Monitoring Board will review and oversee all the source documents and CRFs. The essential information (consent information, enrollment, number and proportion of missed visits, patients lost to follow-up, and AEs) will be monitored and evaluated for completeness and accuracy.

Data will be entered using the double-entry method, and to decrease errors, the data will be checked regularly by the research assistants and overseen by the monitors. Research assistants will double check the data before logging them and will promptly notify the research team if any discrepancies are found. All modifications will be marked on the CRFs. The database will be locked after all the data have been cleaned. If a participant withdraws from the trial either during the treatment period or the follow-up phase, the reasons will be clarified and the rate of participant withdrawal or loss to follow-up will be analyzed statistically.

The study will be monitored by the Scientific Research Department and Ethics Committee of GDPHCM, which is independent of both the investigators and the sponsor. Interim auditing will include off-site surveillance and the submission of a report about the progress of the study.

### Sample size calculation

This study is mainly focused on determining the efficacy of BMF for AR patients with LSQDS. The primary outcome is the TNSS. With reference to data from a previous study [46] and our clinical observations, the sample size was calculated using the noninferiority test for two means with software PASS 2011. We assumed that the TNSS of the treatment group will be 5.53 ± 2.79 and that of the control group will be 6.64 ± 3.06. To obtain a significance level of 5%, a power of 90%, and a noninferiority margin of 0.92, 74 participants are required. Allowing for an 8% dropout rate, 80 participants are needed, with 40 in each group.

### Criteria for stopping treatment

A participant may stop treatment and withdraw from the research project for any reason at any time, and the reason for the withdrawal will be recorded in their CRF. The participants will be told that they have the right to withdraw from the trial and that they will be provided with standardized treatment if they withdraw.

The criteria for stopping treatment and withdrawing from the research project are:

- The participant has suffered an AE related to taking the drug, and the investigator believes it is not appropriate for them to continue taking the drug.
- The participant is diagnosed with asthma or other complications of AR during the study.
- The participant develops another severe disease that needs to be treated during the study.
- Poor compliance, such that the actual drug usage is less than 80% or more than 120% of the prescribed dose.
- Use of drugs proscribed for AR during the study.

### Data analysis

All data analyses will be conducted according to the intention-to-treat principle, and the analysis will be performed in a double-blind manner by qualified statisticians. The EpiData 3.1 software will be used to build the database, and missing data will be replaced with the mean or median of the item for the homogeneous participant population according to complete data. Two similar participants with complete data will be subjected to repeated review, and a logic check will be performed to confirm that the data are correct before analysis.

The data will be analyzed using the Statistical Package for the Social Sciences version 17.0 (SPSS 17.0). Measured data will be expressed as the mean, median, minimum, and maximum values. Comparisons between the two groups will be analyzed by analysis of variance and pairwise comparisons (univariate variance will be assessed with the rank-sum test). Count data will be expressed as the composition ratio and the rate. Between-group efficacy will be assessed using the χ^2^ test, and ranked data will be analyzed with the rank-sum test. A baseline comparison test will be performed at a significance level of α = 0.10, and an efficacy analysis will be performed at a significance level of α = 0.05.

### Ethical approval

The study protocol was approved by the Ethics Committee of GDPHCM (B2016–100-01), and it will be explicitly explained to all participants that the trial involves two types of interventions, with a 2-week run-in period, 4 weeks of treatment and 12 weeks of follow-up. All participants will be given sufficient time to decide whether to sign the informed consent form. Written informed consent must be obtained from each participant before they are randomized to a group.

## Discussion

Although treatment for AR has been developed and standardized, the symptoms of some AR patients are still not well controlled with current pharmacological therapies [47, 48]. CHM, which may have an effect on AR, has gradually received increasing attention and has been adopted for the treatment of AR [8]. Syndrome differentiation or pattern identification is a specific analysis of individual body constitutions, symptoms, and signs based on TCM physiology and pathology and is performed before arriving at a diagnosis or drawing a conclusion [49]. This step must be performed before prescribing CHM formulas because accurate syndrome differentiation or pattern identification ensures accurate therapeutic effects with fewer AEs [8]. BMF is composed of modified *bu zhong yi qi tang* and *yu ping feng san*, which are typically applied to treat allergic diseases [17]. Based on previous experience with the use of BMF to treat AR, this study combines syndrome differentiation and treatment with strict RCT design principles to assess the efficacy and safety of BMF for the treatment of AR. Regarding the mechanistic study of this formulation, this study aims to determine whether BMF can modulate cytokine levels and gut microbiota.

To the best of our knowledge, this is the first double-blind double-dummy RCT aimed at determining the efficacy of BMF in treating AR patients with LSQDS. This is also the first study to investigate changes in inflammatory cytokines as well as changes in the gut microbiota with CHM-based therapy.

There are limitations to this study. The first is a lack of validated outcome measures to evaluate the body constitution of AR patients with LSQDS. In Chinese medicine theory, the body constitution of a patient is dynamic and may change after the BMF intervention. The second limitation is that the sample size calculation was based on a previous study [46] and our clinical observations; thus, the sample size of our study may have been different if more cases had been observed by a prior rigorous RCT. The last limitation is that this study explores only the mechanism by which the BMF formula treats AR and does not address the mechanism at a component level.

Despite its limitations, we believe that this study has the potential to contribute to the development of an effective intervention for AR patients with LSQDS. In the future, a multicenter RCT with a large sample of AR patients and the implementation of multidimensional comprehensive evaluations should be performed. A series of studies on BMF and its chemical components and the molecular mechanisms of actions in AR patients with LSQDS are needed and may provide consolidated evidence regarding the efficacy and safety of BMF for AR patients with LSQDS (Additional file 1).

### Trial status

The first patient in the study was enrolled on 22 December 2016. The trial has already enrolled participants: 76 AR patients have been recruited, and 40 patients had completed the trial by 7 November 2017.

## Additional file

Additional file 1: SPIRIT 2013 checklist. (DOC 149 kb)

## Abbreviations

16S rRNA: 16S ribosomal ribonucleic acid
AE: Adverse event
AR: Allergic rhinitis
BMF: *Bi min fang*
CHM: Chinese herbal medicine
CRF: Case report form
ELISA: Enzyme-linked immunosorbent assay
GDPHCM: Guangdong Provincial Hospital of Chinese Medicine
IFN-γ: Interferon-γ
IgE: Immunoglobulin E
IL-17: Interleukin-17
IL-4: Interleukin-4
LSQDS: Lung and spleen *qi* deficiency syndrome
RCT: Randomized controlled trial
RQLQ(S): Standard version of the Rhinoconjunctivitis Quality of Life Questionnaire
SPSS 17.0: Statistical Packages for the Social Sciences version 17.0
TCM: Traditional Chinese medicine
TGF-β1: Transforming growth factor β-1
TNSS: Total Nasal Symptom Score
VAS: Visual analog scale
